# Time cost of the intraoperative CT in dorsal spinal fusion surgery in degenerative spinal disorders - a retrospective analysis of 518 degenerative cases

**DOI:** 10.1016/j.bas.2026.105993

**Published:** 2026-02-27

**Authors:** Amir Zolal, Jan Mateo, Jörg Silbermann, Milos Paulik, Peggy Gierschner, Matthias Kirsch

**Affiliations:** a Klinik für Neurochirurgie, Klinikum Chemnitz, Germany; b Klinik für Wirbelsäulenchirurgie und Neurotraumatologie, SRH Wald-Klinikum Gera, Germany; c Medizinische Fakultät der TU Dresden, Germany

**Keywords:** Intraoperative CT, Spinal fusion, Degenerative spine, Operative time, Anesthesia time, Surgeon volume

## Abstract

**Introduction:**

To evaluate the impact of intraoperative CT (ioCT)-based navigation on surgery duration, non-surgical anesthesia time, and postoperative length of stay in posterior spinal fusion procedures for degenerative spinal disorders, while accounting for surgeon performance and other procedural factors.

**Research question:**

Does the intraoperative CT use prolong the surgery, and does it affect short term recovery.

**Materials and methods:**

This retrospective, single-center study analyzed 518 cases of dorsal spinal fusion performed with or without ioCT between 2013 and 2018 for degenerative spinal disorders. Demographic and clinical variables such as the number of screws implanted, use of cages, revision status and the surgical volume of the performing surgeon. Surgery duration and postoperative length of stay were assessed using generalized linear models (GLMs).

**Results:**

The mean age of the cohort was 65.67 years (52.5 % female). ioCT-based navigation was employed in 209 of 518 cases (40.3 %). After multivariable adjustment, ioCT increased skin-to-skin time by 38.1 min in the full cohort. The effect persisted in both prespecified subgroups, adding 39.2 min in complex procedures (≥6 screws, ≥4 cages, or iliac screws; n = 233) and 40.2 min in non-complex procedures (n = 285). High-volume surgeons (>50 cases) shortened operative time by ≈ 35 min ioCT showed no significant influence on postoperative length of stay.

**Discussion and conclusion:**

Intraoperative CT-based navigation modestly increases surgical and procedural time. IoCT use did not affect postoperative hospital stay in our analysis, neither improving the short term outcome through better accuracy nor worsening the outcomes through the effects of prolonged surgery time.

## Highlights

• Intraoperative CT (ioCT) increased skin-to-skin time by 38.1 min.
• ioCT also increased non-surgical anesthesia time by 9.36 min.
• High-volume surgeons shortened operative time by approximately 35 min.
• ioCT showed no significant influence on postoperative length of stay (LOS).
• Short-term recovery influenced by complications, not precision of screw placement.

## 1 Introduction

Conflicting reports regarding the influence of intraoperative CT (ioCT) or intraoperative CBCT (cone-beam-CT) based navigation on the surgery duration exist in the present literature. Various authors have reported prolongation of surgery due to the use of this technique (Hecht et al., 2016; Barsa et al., 2014; Baldwin et al., 2022), whereas some reports show no prolongation or even shorter operative times with ioCT/CBCT and navigation, albeit within relatively narrowly defined patient cohorts (Khanna et al., 2016; Knafo et al., 2018). Although various other aspects of the utility of the ioCT/CBCT based navigation of the pedicle screws, like screw placement accuracy and the radiation exposure to the patient and to the surgery team have been explored in depth, the question whether and to what extent the use of this techniques leads to surgery prolongation has not been addressed as a separate research topic. However, the topic of surgery duration is a frequent cause for discussion on the utility of intraoperative imaging and navigation.

Surgery duration is a critical factor in surgical outcomes, as prolonged operative times have been associated with increased risks of postoperative complications, particularly infections. Extended surgeries can lead to greater tissue exposure and longer anesthesia periods, both of which may compromise the patient's immune response and facilitate bacterial contamination. For instance, in spinal surgeries, longer operative times have been linked to higher rates of surgical site infections (Fang et al., 2005).

Therefore, understanding the impact of ioCT or CBCT-based navigation on surgery duration is essential, as any associated prolongation could potentially elevate the risk of postoperative infections and other complications. To explore this topic, we conducted this retrospective, unicentric cohort study on patients with degenerative spinal disorders.

## 2 Methods

### 2.1 Study design and patient population

This retrospective study analyzed patient records from one institution with multiple surgeons involved, spanning the years 2013 to 2018. The initial dataset comprised all patients undergoing spinal surgeries during this period. To focus on degenerative spinal disorders, cases involving traumatic changes, infections or tumor resection were excluded due to differing surgical time requirements. Thus, the final cohort included only patients treated for degenerative spinal pathologies. All patients were treated at (xx anonymized for review).

### 2.2 Inclusion and exclusion criteria

Patients were included in the study if they met the following criteria:

- Underwent spinal fusion surgery between January 1, 2013, and December 31, 2018.
- Diagnosed with degenerative spinal conditions
- Had sufficient surgical records for analysis, including data on ioCT use (if applicable).

Patients were excluded if:

- Their primary diagnosis was trauma, tumor or infectious disease of the spine as the time requirements for resection or reposition differ significantly from degenerative pathologies.
- Cases with incomplete surgical records or missing data on key variables.

### 2.3 Surgical procedures and intraoperative imaging

The surgeries investigated span across the time before and the acquisition of the AIRO ioCT. The use of the AIRO - CT allowed for high-resolution, intraoperative 3D imaging, which facilitated precise navigation in complex degenerative cases. The intraoperative CT (ioCT) was introduced in our institution in 2015; after its installation, ioCT was routinely employed in all spinal fusion procedures unless technical issues or equipment malfunctions prevented its use. No significant changes in imaging protocols were made during the study period. Surgeries that were performed without the use of ioCT were used as a control group.

### 2.4 Data collection and variables

Initially, 6154 patient records of surgeries performed in the specified time period were screened and filtered using the german ICD classification codes for degenerative diseases and surgery codes for posterior instrumented fusion, leading to 550 selected cases. Further filtering was performed during the analysis of the surgery report and the patient record, where further 32 cases were excluded due to erroneous classification or missing data. The resulting dataset thus included 518 cases.

Demographic data (age, sex) were automatically extracted from the patient record, clinical data were extracted manually by analyzing the surgical report and other available data, including: number of screws implanted, use of cages and instrumentation, and ioCT usage. Further variables extracted were: revision surgery (classified as either extension of pre-existing fusion or revision after previous decompressive surgery), use of bone cement, replacement of previously implanted screws (for instance, use of stronger screws or different system), intraoperative replacement of a screw that was deemed malpositioned according to intraoperative fluoroscopy, dural tear, use of iliac screws and harvesting of pelvic crest bone.

### 2.5 Statistical analysis

The primary statistical analysis focused on evaluating the influence of ioCT on three key outcome variables: operative time (skin-to-skin), non-surgical anesthesia time (total anesthesia duration minus skin-to-skin time), and postoperative length of stay (LOS). A generalized linear model (GLM) was used for these analyses, incorporating ioCT usage, surgeon case volume (categorized as high-volume surgeons with more than 50 surgeries versus others), number of implanted screws and cages, number of decompressed segments, and various surgical and anatomical factors.

Patients were further stratified into two clearly defined subgroups: complex and other. Complex procedures were defined as those involving six or more screws, four or more cages, or the use of iliac screws. The rest of the cohort was included in the non-complex subgroup. The sub-grouping variable was chosen arbitrarily to maintain a fairly even distribution of cases between complex and non-complex groups (approximately 100 CT cases were assigned to each subgroup) using these criteria. Automated stepwise variable selection based on the Akaike Information Criterion (AIC) was performed independently within each subgroup and on the entire dataset to identify optimal predictive models.

To assess whether a learning effect occurred after the introduction of intraoperative CT (ioCT) in 2015, we performed a time-trend analysis restricted to cases with ioCT use. The date of surgery was used to calculate the number of months elapsed since ioCT introduction (January 2015). Skin-to-skin time was modeled as a function of this variable using ordinary least squares (OLS) regression. An initial unadjusted model (SNZ ∼ months since introduction) was followed by an adjusted model that included the same case-mix covariates used in the multivariable analysis (cages, number of screws, decompressed segments, spinal level, dural tear, cement augmentation, revision status, and minimally invasive approach). To further explore whether the effect was concentrated in the early adoption phase, a piecewise regression model with separate slopes for the first 12 months and for later months was fitted.

To evaluate interobserver agreement, surgical records of 210 randomly selected cases were reviewed independently by one doctoral candidate and one attending neurosurgeon. Variables assessed included the number of screws, cages, decompressed segments, revision status, revision type (after decompression or adjacent segment), intraoperative screw repositioning, spinal region involvement (cervical, thoracic, lumbar), use of iliac screws, and iliac crest bone harvesting. Intraclass correlation coefficients were calculated for these variables to quantify interobserver reliability.

All analyses were conducted using R software (version 4.4.2). Statistical significance was set at p < 0.05.

## 3 Results

Baseline characteristics of the 518 included patients are shown in Table 1. A total of 206 patients (39.8%) underwent surgery with intraoperative CT (ioCT), while 312 (60.2%) were treated without ioCT. The mean age was 65.7 years (SD 11.9), and sex distribution was similar between groups (47.5% male overall, p = 0.705, χ^2^). In contrast, operative and anesthesia-related times were significantly longer in the ioCT group: mean skin-to-skin time, anesthesia duration, and non-surgical anesthesia time were all prolonged compared with the non-ioCT group (all p < 0.001, *t*-test). Postoperative length of stay did not differ significantly (p = 0.112, *t*-test).

**Table 1:** Baseline demographic and surgical characteristics of patients with and without intraoperative CT (ioCT). Values are presented as mean ± SD, median [IQR], or n (%). Between-group differences were tested using *t*-test for normally distributed continuous variables, Mann–Whitney *U* test for non-normally distributed variables, and χ^2^ test for categorical variables.

Variable	No-CT	CT	Total	p-value
Age (years), mean ± SD	64.9 ± 11.9	66.9 ± 11.9	65.7 ± 11.9	0.057 (*t*-test)
Male, n (%)	154 (49.4%)	92 (44.7%)	246 (47.5%)	0.338 (Chi^2^)
Skin-to-Skin time (min), mean ± SD [range]	191.3 ± 60.4 [77-464]	233.5 ± 75.7 [89-479]	208.1 ± 70.0 [77-479]	**<0.001 (*t*-test**)
Non-surgical anesthesia (min), mean ± SD [range]	67.7 ± 18.3 [-94-149]	78.4 ± 22.7 [21-175]	72.0 ± 20.8 [-94-175]	**<0.001 (*t*-test)**
Postoperative length-of-stay (days), mean ± SD [range]	8.8 ± 6.2 [3-44]	9.0 ± 6.9 [3-65]	8.9 ± 6.5 [3-65]	0.749 (*t*-test)
Screws, median [IQR]	4 [4-6]	5 [4-6]	4 [4-6]	**<0.001 (Mann-Whitney U)**
Cages, median [IQR]	1 [1-2]	1 [1-2]	1 [1-2]	0.257 (Mann-Whitney U)
Iliac screw (n)	7	11	18	0.101 (Chi^2^)
Minimal invasive (n)	16	6	22	0.317 (Chi^2^)
Dural tear (n)	50	25	75	0.270 (Chi^2^)
Revision surgery (n)	99	41	140	**0.004 (Chi^2^)**
High-volume surgeon (n)	219	145	364	1.000 (Chi^2^)

Out of eight main surgeons identified in the records, three have performed more than 50 surgeries in the study group and were included in the high - volume group.

The numbers of implanted screws and cages were comparable between groups (p = 0.152 and p = 0.128, Mann–Whitney U). Similarly, rates of iliac screw use, minimally invasive procedures, dural tears, and revision surgeries showed no significant differences. The proportion of operations performed by high-volume surgeons was also balanced between groups (p = 0.644, χ^2^).

**Fig. 1:**
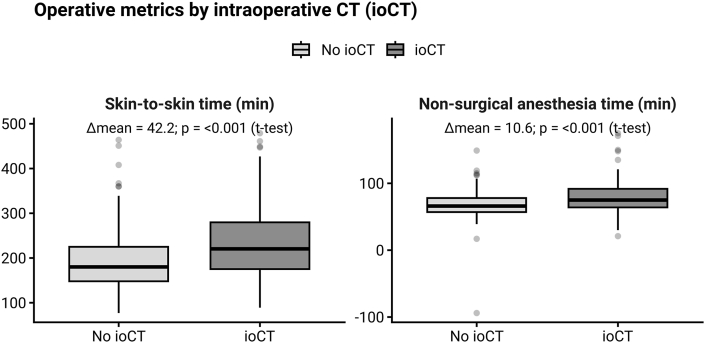
Skin-to-skin and non-surgical anesthesia times in cases with versus without intraoperative CT (ioCT), with annotated mean differences and p-values (Welch's *t*-test).

Taken together, the ioCT and non-ioCT cohorts were broadly comparable in demographics and surgical complexity, with the main baseline difference being longer operative and anesthesia times in the ioCT group. The univariate analyses of the differences of skin-to-skin time and the non-surgical anesthesia time between the ioCT and no-CT group are also illustrated in Fig. 1.

### 3.1 Interobserver agreement

For the interobserver variable comparison, the 14 variables that were assessed manually by two observers were pooled for an intraclass correlation coefficient analysis. The pooled intraclass correlation coefficient (ICC) for all 14 variables was 0.95 (95% CI: 0.94–0.95) for single random raters, indicating excellent interobserver reliability. When averaged across raters, the ICC increased to 0.97 (95% CI: 0.97–0.98), with a statistically significant result (F = 37, p < 0.001).

### 3.2 Influence on the skin-to-skin time

**Fig. 2:**
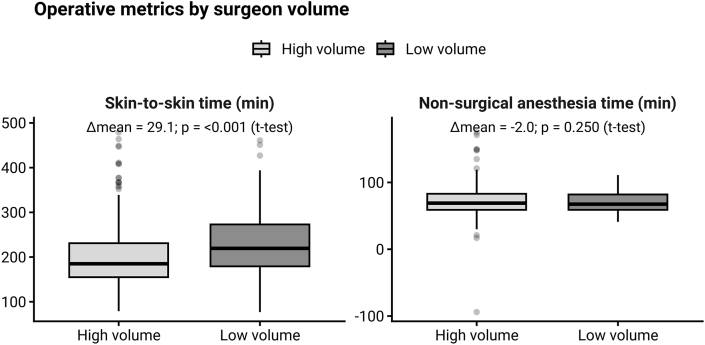
Skin-to-skin and non-surgical anesthesia times for high-volume (Silbermann, Paulik, Schwerter) versus low-volume surgeons, with annotated mean differences and p-values (Welch's *t*-test).

Table 3 presents the GLM results for skin-to-skin time across the full cohort and within the two subgroups (Complex vs. Other). In the full dataset model, intraoperative CT (ioCT) emerged as a highly significant predictor, increasing skin-to-skin time by an estimated 38.1 min (p < 0.001) after adjustment for surgeon volume, screw and cage counts, decompressed segments, and other surgical factors. High-volume surgeons (those with >50 cases) demonstrated on average 35.0 min shorter skin-to-skin time than lower-volume surgeons (p < 0.001). As this factor emerged as highly significant, univariate analyses of the influence of surgeon experience on surgery duration are included in Table 2. The resulting boxplots are shown in Fig. 2.

**Table 2:** Univariate analyses of the differences in the influence of high volume vs. low volume surgeon.

Variable	High volume (n = 364)	Low volume (n = 154)	p-value (*t*-test)
Skin-to-skin time [min]	199.4 ± 67.8	228.5 ± 70.9	<0.001
Non-surgical anesthesia time [min]	72.5 ± 22.7	70.6 ± 15.5	0.250
Hospital stay [days]	9.1 ± 6.7	8.4 ± 6.1	0.254

**Table 3:** Results of the statistical analysis for the skin-to-skin time.

Variable	Estimate [minutes]	Std. Error	t value	p value	Cases (CT/No CT)
**Full group analysis**					
intraoperative CT scan [yes/no]	38.1	5.0	7.575	<0.001	206/312
high volume surgeon (>50 cases in database)	−35.0	5.2	−6.661	<0.001	
Cages [n]	40.0	4.2	9.450	<0.001	
Decompressed segments [n]	13.4	3.5	3.790	<0.001	
Dural tear [yes/no]	19.2	5.7	3.358	<0.001	
Cement [yes/no]	−2.4	1.4	−1.766	0.079	
Replacement of previously implanted screws (same position) [yes/no]	4.7	2.8	1.644	0.101	
Thoracic spine involved [yes/no]	49.6	11.0	4.519	<0.001	
Cervical spine involved [yes/no]	47.4	14.4	3.297	0.001	
Iliac screws [yes/no]	42.5	13.5	3.154	0.002	
Iliac crest harvested [yes/no]	32.1	8.0	3.994	<0.001	
Revision surgery [yes/no]	−12.8	8.6	−1.488	0.137	
Replacement of screws in same surgery [yes/no]	19.3	5.8	3.324	<0.001	
Revision surgery because of adjacent segment [yes/no]	32.0	11.0	2.911	0.004	
**Subgroup analysis - complex procedures (**≥6 screws, ≥4 cages, or iliac screws)					
intraoperative CT scan [yes/no]	39.2	8.7	4.490	<0.001	104/129
high volume surgeon (>50 cases in database)	−34.8	9.5	−3.660	<0.001	
Cages [n]	29.8	7.0	4.275	<0.001	
Decompressed segments [n]	9.3	4.6	2.007	0.040	
Dural tear [yes/no]	13.2	9.5	1.389	0.100	
lumbar spine involved [yes/no]	47.7	27.6	1.727	0.091	
thoracic spine involved [yes/no]	32.0	18.1	1.766	0.082	
cervical spine involved [yes/no]	72.7	28.8	2.523	0.013	
iliac screws [yes/no]	29.1	17.1	1.703	0.090	
iliac crest harvesting [yes/no]	49.9	12.6	3.956	<0.001	
Revision [yes/no]	−82.5	46.2	−1.787	0.075	
Replacement of screws in same surgery [yes/no]	15.8	8.4	1.883	0.061	
Revision after non-instrumented decompression surgery [yes/no]	77.2	48.7	1.586	0.114	
Revision because of adjacent disease [yes/no]	114.4	47.5	2.407	0.017	
**Subgroup analysis - non-complex procedures (**<6 screws, <4 cages, *no* iliac screws)					
intraoperative CT scan [yes/no]	40.2	5.5	7.280	<0.001	102/183
high volume surgeon (>50 cases in database)	−32.8	5.7	−5.774	<0.001	
cages [n]	32.0	9.2	3.472	<0.001	
decompressed segments [n]	24.0	8.1	2.955	0.003	
dural tear [yes/no]	22.0	6.6	3.314	1.04e-03	
cement [yes/no]	−3.4	2.1	−1.626	0.105	
thoracic spine involved [yes/no]	71.6	17.0	4.218	<0.001	
Replacement of screws in same surgery [yes/no]	24.0	8.9	2.685	0.008	
Revision because of adjacent disease [yes/no]	18.1	7.0	2.599	0.098

Regarding a possible learning curve effect after the introduction of the ioCT, the ioCT subgroup (n = 206; June 2015–December 2018) was analyzed. The skin-to-skin time showed a non-significant trend toward reduction over time. In the unadjusted model, operative time decreased by −0.6 min per month (≈−7 min per year, p = 0.16). Adjustment for case-mix yielded a similar slope (−0.6 min per month, p = 0.13). Piecewise modeling suggested a steeper decline during the first 12 months after introduction (−4.5 min per month, ≈ −54 min per year), followed by a flatter slope thereafter (−0.3 min per month), although neither reached statistical significance (p = 0.21 and p = 0.50, respectively).

Within the complex-case subgroup (n = 233, from that 104 with ioCT and 129 without ioCT, 6 or more screws, 4 or more cages or iliac screws use), ioCT remained a significant contributor to longer skin-to-skin time, adding 39.2 min on average (p < 0.001). Other significant covariates in this group included surgeon volume (high-volume vs. other, – 34.8 min, p < 0.001), number of screws and cages, and revision status.

In the non-complex subgroup (n = 285, from that 102 with ioCT and 183 without ioCT), ioCT again significantly prolonged the surgery by 40.2 min (p < 0.001). High-volume surgeons were faster (– 32.8 min, p < 0.001). Additional independent predictors included the numbers of screws and cages, decompressed segments, and intraoperative screw repositioning.

Overall, these results confirm that ioCT adds a consistent time cost to posterior instrumented fusion procedures—on the order of 38–40 min—regardless of case complexity, while high surgical volume of the surgeon mitigates the surgery duration across all settings.

### 3.3 Influence on the non-surgical anesthesia time (skin-to skin subtracted from anesthesia duration)

In the full-cohort GLM (Table 4), ioCT usage was independently associated with an increase of **9.4 min** in non-surgical anesthesia time (p < 0.001). Procedural complexity variables—including the number of cages, cement augmentation, and involvement of the thoracic or cervical spine—also remained significant predictors, whereas surgeon case volume and revision status were not retained in the final model. This underscores that ioCT contributes a measurable anesthesia overhead even after accounting for key surgical factors.

**Table 4:** Influence on the non-surgical anesthesia time (skin-to-skin subtracted from anesthesia time).

Variable	Estimate [minutes]	Std. Error	t value	p value
intraoperative CT scan [yes/no]	9.4	1.7	5.463	<0.001
cages [n]	4.9	1.3	3.801	<0.001
cement [n]	−1.1	0.5	−2.391	0.017
thoracic spine involved [yes/no]	7.8	3.7	2.140	0.033
cervical spine involved [yes/no]	32.0	4.7	6.825	<0.001
iliac screws [yes/no]	22.2	4.6	4.878	<0.001
iliac crest harvested [yes/no]	4.5	2.8	1.627	0.104

### 3.4 Influence on the postoperative length of stay

Table 5 presents the GLM results for postoperative length of stay (LOS) in the full cohort. Unlike skin-to-skin and anesthesia times, ioCT usage did not remain a significant predictor of LOS after stepwise selection. The final model instead included number of decompressed segments (0.6 days per level, p = 0.068), presence of an intraoperative dural tear (3 days, p < 0.001), intraoperative screw repositioning (0.7 days, p = 0.006), thoracic spine involvement (3.8 days, p < 0.001), cervical spine involvement (6.4 days, p < 0.001), and iliac screw placement (– 1.9 days, p = 0.040). These results indicate that surgical complexity and intraoperative complications, rather than ioCT navigation, primarily determine postoperative LOS.

**Table 5:** Influence on the postoperative length-of-stay.

Variable	Estimate [days]	Std. Error	t value	p value
Decompressed segments [n]	0.6	0.3	1.827	0.068
Dural tear [yes/no]	3.0	0.6	4.745	<0.001
Replacement of previously implanted screws (same position) [yes/no]	0.7	0.3	2.749	0.006
thoracic spine involved [yes/no]	3.8	1.1	3.427	<0.001
cervical spine involved [yes/no]	6.4	1.5	4.372	<0.001
iliac crest harvested [yes/no]	−1.9	0.9	−2.059	0.040

## 4 Discussion

This study analyzed the impact of intraoperative CT (ioCT)-based navigation on operative duration, non-surgical anesthesia time, and postoperative length of stay (LOS) in patients undergoing posterior spinal fusion for degenerative spinal disorders. The use of ioCT consistently contributed to a significant increase in operative duration across the entire cohort, adding approximately 38 min to skin-to-skin time. Notably, surgeon experience also emerged as a significant factor, with high-volume surgeons (defined as those performing more than 50 surgeries during the study period) reducing operative time by approximately 35 min on average.

In contrast to operative duration, the increase in non-surgical anesthesia time attributable to ioCT was comparatively modest but still statistically significant, adding approximately 9 min. This increase likely reflects additional preparation time required for navigation setup and image acquisition procedures. Other significant contributors to increased non-surgical anesthesia time included the involvement of the cervical or thoracic spine and surgeries requiring iliac fixation. Surgeon experience was not a significant predictor in this context, suggesting that anesthesia-related workflows are less affected by individual surgeon variability.

Interestingly, ioCT usage did not significantly impact postoperative LOS. Instead, postoperative hospital stay duration was more strongly influenced by intraoperative complications such as dural tears, intraoperative screw repositioning, and the number of decompressed spinal segments. These results indicate that although ioCT contributes to extended operative and anesthesia durations, it does not adversely affect short-term postoperative recovery, as reflected in LOS. Although ioCT facilitates more accurate intraoperative screw placement, this increased precision did not translate into measurable improvements in the immediate postoperative course. These findings suggest that short-term recovery is primarily influenced by clinical factors and intraoperative complications rather than the accuracy of screw placement alone.

Previous studies have consistently demonstrated higher screw placement accuracy using ioCT/CBCT-based navigation, particularly regarding intrapedicular screw positioning (Van de et al., 2012; La et al., 2022) and reduced facet joint violations (Yson et al., 2013). This technique offers multiple advantages, including lower radiation exposure for the surgical team (Abdullah et al., 2012; Bandela et al., 2013) and improved cost-effectiveness due to a decreased need for reoperations (Sanborn et al., 2012). A similar effect was observed in fractures (Brunken et al., 2024), where navigation led to higher screw implantation accuracy and to the use of larger-diameter screws with possible implications for implant stability (Mandelka et al., 2023).

The accuracy of pedicle screw placement with navigation has been investigated extensively across many studies, including multiple systematic reviews/meta-analyses synthesizing tens of thousands of screws (Baldwin et al., 2022; Tian et al., 2011; Staartjes et al., 2018; Aoude et al., 2015; Gelalis et al., 2012; Papalia et al., 2024). A recent meta-analysis pooled 30 studies with 17,911 patients and 24,600 screws and confirmed higher clinically acceptable accuracy with navigation (96.2% vs 94.2%) (Papalia et al., 2024), while a methodological review covering 68 studies and 43,305 screws showed 97.3% vs 91.4% within a <2 mm breach threshold for navigated versus non-navigated placement (Aoude et al., 2015). Accordingly, our study focused on a different clinically relevant question—time cost and workflow impact of intraoperative CT—rather than re-quantifying accuracy.

The influence of ioCT navigation on operative time remains debated. Some studies report increased durations compared to conventional fluoroscopy (Hecht et al., 2016; Barsa et al., 2014), while others, such as Khanna et al., observed shorter durations (Khanna et al., 2016). Such discrepancies might reflect variability in surgeon expertise, experience with navigation systems, and procedural nuances rather than intrinsic limitations of navigation technology itself.

Our findings underscore the critical role surgeon experience plays in operative efficiency. Prior research by Khanna et al. (2016) and Knafo et al. (2018) similarly emphasized that individual surgeon proficiency strongly influences operative duration, potentially offsetting navigation-related delays.

Several limitations should be acknowledged. This was a retrospective, single-center study, potentially limiting generalizability. Although comprehensive, our subgroup definitions may not fully encompass procedural variability. Additionally, despite high interobserver reliability in our data extraction process, retrospective manual data collection could introduce observer bias. As the importance of surgeon experience is a key finding, this may further limit generalizability, since our high-volume surgeons' results may not fully translate to centers with different case distributions or experience levels. While we employed generalized linear models with stepwise selection to identify significant factors, the large number of variables and tests increases the risk of false positives, as corrections for multiple comparisons were not applied; therefore, results should be interpreted with appropriate caution. Moreover, our dataset included cases from the introductory period of ioCT implementation, during which longer procedure times could be expected due to the learning curve. To address this, we performed a dedicated statistical analysis of operative times over the study period. This analysis did not demonstrate a significant learning-curve effect, and the non-significant trends that were observed were of smaller magnitude than the overall effect associated with ioCT use. Future studies should ideally involve prospectively defined, standardized surgical cohorts to further clarify ioCT's clinical impact, particularly regarding long-term patient outcomes beyond immediate postoperative parameters.

## 5 Conclusion

This study provides valuable insights into the impact of ioCT-based navigation on surgical workflow in dorsal spinal fusion for degenerative spinal disorders. While ioCT was associated with moderate increases in operative and non-surgical anesthesia time, its influence was comparable to surgeon performance in general. Importantly, the use of ioCT navigation neither negatively impacted nor improved short-term recovery outcomes, as evidenced by its lack of significant effect on postoperative length of stay. Although ioCT ensures greater accuracy in screw placement, this precision alone did not lead to measurable improvements in the immediate postoperative recovery, suggesting that short-term patient outcomes are more strongly influenced by clinical factors and intraoperative complications than by navigational precision alone.

## Declaration of competing interest

The authors have declared no conflict of interest.
